# Evolution of impedance field telemetry after one day of activation in cochlear implant recipients

**DOI:** 10.1371/journal.pone.0173367

**Published:** 2017-03-06

**Authors:** Hao-Chun Hu, Joshua Kuang-Chao Chen, Chia-Mi Tsai, Hsing-Yi Chen, Tao-Hsin Tung, Lieber Po-Hung Li

**Affiliations:** 1 Department of Otolaryngology, Cheng Hsin General Hospital, Taipei, Taiwan; 2 Institute of Clinical Medicine, National Yang-Ming University, Taipei, Taiwan; 3 Cochlear Implant Center, Far Eastern Memorial Hospital, New Taipei, Taiwan; 4 Faculty of Medicine, School of Medicine, National Yang-Ming University, Taipei, Taiwan; 5 Department of speech language pathology and audiology, National Taipei University of Nursing and Health Sciences, Taipei, Taiwan; 6 Department of Otolaryngology, Lotung Poh-Ai Hospital, Yilan, Taiwan; 7 Department of Medical Research and Education, Cheng Hsin General Hospital, Taipei, Taiwan; 8 Faculty of Public Health, School of Medicine, Fu-Jen Catholic University, Taipei, Taiwan; University of California Irvine, UNITED STATES

## Abstract

**Objectives:**

Changes in impedance between 24 hours and one month after cochlear implantation have never been explored due to the inability to switch on within one day. This study examined the effect of early activation (within 24 hours) on the evolution of electrode impedance with the aim of providing information on the tissue-to-electrode interface when electrical stimulation was commenced one day post implantation.

**Methods:**

We performed a retrospective review at a single institution. Patients who received a Nucleus 24RECA implant system (Cochlear, Sydney, Australia) and underwent initial switch-on within 24 hours postoperatively were included. Impedance measurements were obtained intraoperatively and postoperatively at 1 day, 1 week, 4 weeks, and 8 weeks.

**Results:**

A significant drop in impedance was noted 1 day after an initial activation within 24 hours followed by a significant rise in impedance in all channels until 1 week, after which the impedance behaved differently in different segments. Basal and mid-portion electrodes revealed a slight increase while apical electrodes showed a slight decrease in impedance from 1 week to 8 weeks postoperatively. Impedance was relatively stable 4 weeks after surgery.

**Conclusions:**

This is the first study to report the evolution of impedance in all channels between initial mapping 1 day and 1 month after cochlear implantation. The underlying mechanism for the differences in behavior between different segments of the electrode may be associated with the combined effect of dynamics among the interplay of cell cover formation, electrical stimulation, and fibrotic reaction.

## Introduction

A cochlear implant (CI) is a surgically implanted prosthetic device that electrically stimulates the cochlear nerve to provide hearing. Cochlear implantation is an important surgical technique for patients with severe to profound sensorineural hearing loss, and even in patients with inner ear malformation [1, 2]. Hearing/speech performance and music perception can be improved after surgery [3, 4].

During implantation and mapping, various parameters are used to examine the integrity of the device, of which impedance field telemetry is the most commonly used. Changes in impedance related to the tissue-to-electrode interface and surrounding environment of the cochlea have been reported in several studies [5–8]. In our previous study, we found a significant drop in impedance during initial mapping within 24 hours after cochlear implantation which may have been related to spontaneous recovery of the micro-environment inside the cochlea and a divergence effect of electrical stimulation after the device had been switched on [9]. Changes in impedance after early activation have also been reported in other studies [10, 11]. However, the detailed evolution of impedance after 1 day of activation has not previously been investigated.

In this study, we examined the effect of early activation (within 24 hours) on the evolution of electrode impedance through a chart review of patients receiving cochlear implantation. The aim of this study was to provide information on the tissue-to-electrode interface when electrical stimulation was commenced 1 day post implantation.

## Methods

All patients who received a Nucleus 24RECA implant system (Cochlear, Sydney, Australia) at Cheng Hsin General Hospital and who underwent initial switch-on within 24 hours postoperatively were included. This study included 16 male and 14 female patients (median age, 18 years; age range, 2 to 79 years). Of the 30 patients, 16 received a CI on the right side and 14 on the left side. This retrospective chart review study was approved by the Institutional Ethics and Research Committee of Cheng Hsin General Hospital and waived the requirement for informed consent.

### Surgical procedures

The surgical technique with a small incision for cochlear implantation that we use in our department has been described in detail in our previous studies [9, 12]. In brief, the surgery involved a 2.5- to 3-cm postauricular incision, drilling of a bone housing for the receiver-stimulator, harvesting of a cortex bone chip from the mastoid using minimal mastoidectomy, posterior tympanostomy, and hyaluronic acid gel coverage of the cochleostomy before array insertion. A soft technique with the Advance Off-Stylet technique was used for insertion in all cases [13, 14]. After insertion, defects of the mastoid cavity were sheltered by the harvested bone chip, and the wound was sutured layer by layer. Full insertion of the electrode array was confirmed in each patient postoperatively by X-ray.

### Measurements

The speech processor was switched on 1 day postoperatively. The default settings during switch-on included a 25-ms pulse width, 900 pps rate, and Advanced Combination Encoder strategy. Impedance (kOhm) was measured from Channel (CH) 1 to CH 22 using Custom Sound EP software (version 3.2, Cochlear, Sydney, Australia). Impedance measurements were performed intraoperatively and postoperatively at 1 day, 1 week, 4 weeks, and 8 weeks in both common ground and monopolar modes (MP1 mode, MP2 mode, and MP1+2 mode).

### Statistics

All statistical analyses were performed using SPSS version 18.0.0 (SPSS, Inc., Chicago, IL, US). A paired sample t-test was used to compare values from consecutive fitting sessions. Continuous data were presented as mean ± standard deviation (SD). Statistical significance was set at P ≤ 0.05.

## Results

**Fig 1:**
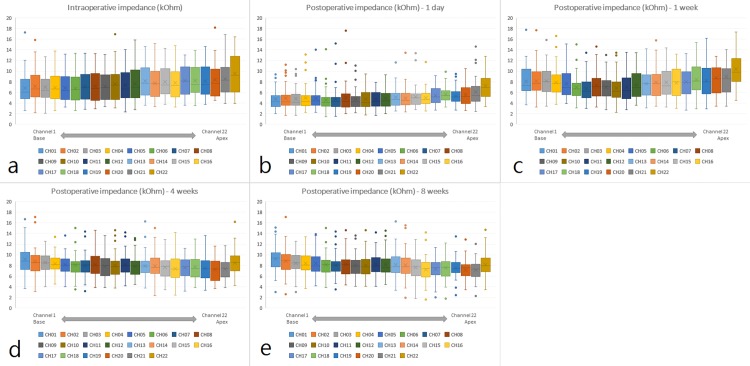
Impedance measurements for each of the 22 electrodes. Median values are displayed as horizontal lines and mean values as crosses. Circles denote extreme values. (a) Intraoperative measurements, (b) 1 day after surgery, (c) 1 week after surgery, (d) 4 weeks after surgery, (e) 8 weeks after surgery.

**Fig 2:**
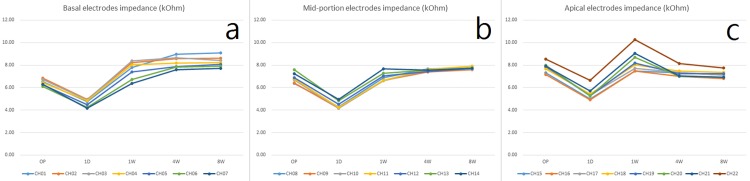
Evolution of electrodes impedance over 8 weeks. (a) Basal electrodes, (b) Mid-portion electrodes, (c) Apical electrodes.

**Fig 3:**
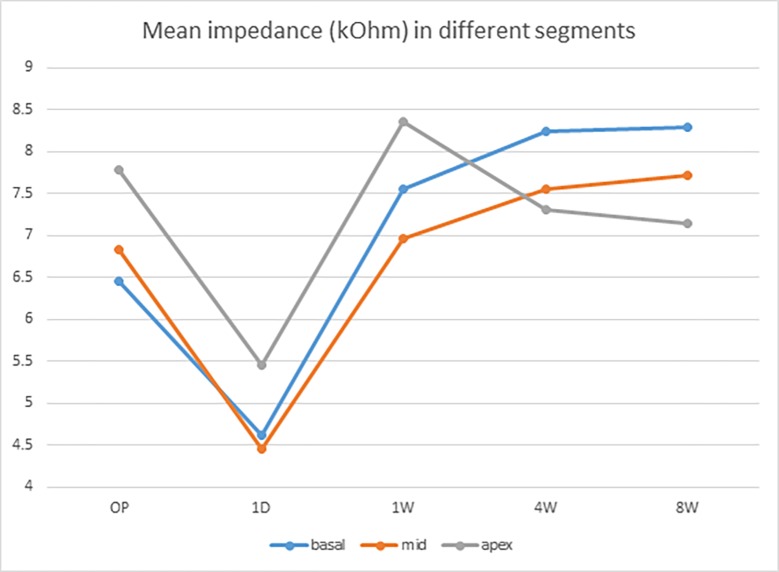
Evolution of mean impedance of the apical, mid-portion and apical electrodes over 8 weeks.

We defined basal electrodes as CH 1 to CH 7, mid-portion electrodes as CH 8 to CH 14, and apical electrodes as CH 15 to CH 22 [8, 15, 16] and MP2 mode impedance values (S1 File) were analyzed. A significant drop in mean impedance 1 day after cochlear implantation was noted in all channels (Figs 1 and 2) (Table 1). One week after the surgery, a significant rise in mean impedance was noted in all channels, after which the impedance behaved differently in different segments. A slight increase in impedance was noted in the basal and mid-portion electrodes with a slight decrease in the apical electrodes from 1 week to 8 weeks (Figs 2 and 3). Differences in impedance were significant in all channels from intraoperative measurements to 1 day and from 1 day to 1 week (Table 2). The changes from 1 week to 4 weeks were significant in CH 1, CH 6, CH 7, CH 9, CH 10, CH 11, CH 20, CH 21, and CH 22 (Table 2), however there were no significant changes from 4 weeks to 8 weeks (Table 2). In addition, no short or open circuits of the devices were noted intraoperatively or postoperatively. We also analyzed common ground mode impedance values. In general, common ground mode produced 13.3% lower impedance than MP2 mode, however the significance of impedance evolution did not change.

**Table 1:** Results of Mean Impedance Values (kOhm) in All Channels.

		*OP*	*1D*	*1W*	*4W*	*8W*
*Basal electrodes*	CH01	6.86	5.29	8.29	9.5	9.75
	CH02	7.33	5.72	8.92	9.27	9.48
	CH03	7.22	5.77	9.2	9.47	9.39
	CH04	7.18	5.79	8.96	9.1	9.28
	CH05	7.04	5.5	8.44	8.88	9.16
	CH06	6.94	5.34	7.81	8.88	9.07
	CH07	7.28	5.32	7.52	8.69	9.02
	Mean	7.12	5.53	8.45	9.11	9.31
	(SD)	-2.39	-1.86	-2.76	-1.85	-1.93
*Mid-portion* e*lectrodes*	CH08	7.27	5.45	8.04	8.81	9
	CH09	7.34	5.38	7.82	8.54	8.88
	CH10	7.61	5.39	7.81	8.74	8.92
	CH11	7.72	5.48	7.88	8.84	9.24
	CH12	7.82	5.73	8.28	8.56	9.02
	CH13	8.57	6.06	8.51	8.77	9.04
	CH14	8.13	6.18	8.89	8.78	9.04
	Mean	7.78	5.67	8.17	8.72	9.02
	(SD)	-2.52	-1.75	-2.46	-1.87	-1.99
*Apical electrodes*	CH15	8.22	6.13	8.69	8.5	8.43
	CH16	8.15	6.11	8.7	8.2	8.11
	CH17	8.76	6.52	8.89	8.53	8.42
	CH18	8.65	6.61	9.2	8.66	8.65
	CH19	8.87	6.43	9.37	8.42	8.52
	CH20	8.92	6.46	9.91	8.25	8.26
	CH21	8.89	6.87	10.23	8.26	8.21
	CH22	9.43	7.8	11.4	9.3	8.99
	Mean	8.74	6.62	9.55	8.51	8.45
	(SD)	-2.74	-1.99	-3.4	-2.06	-1.88

**Table 2:** Comparison of Impedances between Different Measurement Times.

		*OP vs*. *1D*	*1D vs*. *1W*	*1W vs*. *4W*	*4W vs*. *8W*
*Basal electrodes*	CH01	0.001*	<0.001*	0.001*	0.332
	CH02	<0.001*	<0.001*	0.396	0.295
	CH03	0.004*	<0.001*	0.524	0.73
	CH04	0.001*	<0.001*	0.78	0.325
	CH05	<0.001*	<0.001*	0.371	0.126
	CH06	<0.001*	<0.001*	0.006*	0.362
	CH07	<0.001*	<0.001*	0.002*	0.179
*Mid-portion* e*lectrodes*	CH08	<0.001*	<0.001*	0.064	0.519
	CH09	<0.001*	<0.001*	0.029*	0.325
	CH10	<0.001*	<0.001*	0.029*	0.521
	CH11	<0.001*	<0.001*	0.018*	0.2
	CH12	<0.001*	<0.001*	0.459	0.148
	CH13	<0.001*	<0.001*	0.536	0.397
	CH14	<0.001*	<0.001*	0.82	0.362
*Apical electrodes*	CH15	<0.001*	<0.001*	0.984	0.463
	CH16	<0.001*	<0.001*	0.21	0.685
	CH17	<0.001*	<0.001*	0.393	0.615
	CH18	<0.001*	<0.001*	0.294	0.965
	CH19	<0.001*	<0.001*	0.087	0.713
	CH20	<0.001*	<0.001*	0.034*	0.973
	CH21	<0.001*	<0.001*	0.044*	0.867
	CH22	<0.001*	<0.001*	0.01*	0.281

*Statistical significance was defined as P ≤ 0.05

## Discussion

Advances in minimally invasive CI surgery in recent years has led to less postoperative pain and swelling, making earlier device activation possible [17]. Traditionally, the earliest activation time was 4 to 6 weeks after surgery. However, various studies investigating early activation have been published and shown the safety, feasibility and residual hearing preservation [9–12, 18, 19]. Early activation within 24 hours after surgery has been reported by our hospital [9, 12] and others [10]. A minimal access approach is critical to facilitate initial switch-on within 1 day, as this can help to minimize the degree of postoperative swelling [12]. In our series, all of the patients tolerated early activation without any complications. However, the detailed evolution of impedance after early electrical stimulation within 24 hours has not previously been reported.

### Mode of stimulation

The Nucleus 24RECA implant system allows for impedance to be measured in common ground or monopolar modes. Common ground mode uses one intracochlear electrode as the active electrode and shorts the other electrodes on the array as the return. In monopolar mode, one intracochlear electrode is activated, and one or both extracochlear electrodes are chosen as the return electrode: MP1 mode (ball), MP2 mode (plate) and MP1+2 mode (plate and ball) [8]. Thus, the monopolar mode can better illustrate changes in any individual intracochlear electrode impedance over time, and therefore we analyzed MP2 mode impedance values in this study. On the other hand, impedance has been shown to be inversely related to the geometric surface area of the electrodes [6], and there is a larger surface area of the return electrode in common ground mode than in monopolar mode [20]. Therefore, impedance values are lower in common ground mode.

### Intraoperative measurements

In this study, the intraoperative measurements showed that the apical electrodes had the highest values while the basal electrodes had the lowest values (Fig 1) (Table 1), which is compatible with previous studies [20, 21]. In addition, impedance values are inversely related to the geometric surface area of the electrodes [6]. For the Nucleus 24RECA implant system, the active geometric surface of the electrodes is reduced in a basal-to-apical direction, which in turn can lead to higher impedance values for the apical electrodes.

### One-day drop

In our series, an acute drop in impedance was seen in all channels 1 day after surgery (Figs 2 and 3), as first reported in one of our previous studies [9]. A possible explanation for this is rearrangement of the milieus surrounding the electrode array after surgery [19]. For example, air bubbles generated by electrode insertion may have led to high intraoperative impedance values which then resolved quickly after implantation [18]. Another possible explanation for this 1-day drop is a divergence (blow-out) effect due to initial electrical stimulation after activation [9]. A cell cover composed of protein adsorption, macrophages and fibroblasts will form around the electrodes within hours after insertion, and this will lead to increased impedance [7]. Electrical stimulation after switch-on may then trigger cell escape from the surface of the electrode array [7, 8]. A hydride layer would then be formed, which in turn could increase the contact area of the electrodes leading to a reduction in impedance [22].

### Evolution of impedance from 1 day to 4 weeks

The second significant finding in our series was the acute rise of impedance in all channels from 1 day to 1 week postoperatively. During this period, the impedance behaved differently in different segments, with a slight increase in impedance in basal and mid-portion electrodes and a slight decrease in apical electrodes (Fig 3). Several studies have reported that this evolution of impedance may be affected by intraoperative steroid application [19, 23], however we did not use steroids in our patients at any time point. The trend of differences in impedance in different segments may be associated with factors including the composition of impedance and the effect of electrical stimulation.

#### Composition of impedance: Cell cover formation and fibrosis

The measured impedance depends on the results of interplay between the degree of cell cover formation and fibrosis on site [5, 6]. Cell cover formation begins within hours after cochlear implantation, while fibrosis starts from 2 to 5 days after wounding resulting in a gradual rise in impedance [5, 8, 11, 19, 24]. Tykocinski et al. also reported that cell cover formation by protein and immune cells had a more prominent effect on changes in impedance than fibrosis within the initial few weeks postoperatively [6].

#### Effect of electrical stimulation

The electrical stimulation applied after activation has been reported to affect the electrode surface rather than create changes in the surrounding tissue (i.e. fibrotic reaction) [6]. It is thus reasonable to infer that the course of fibrosis is relatively unaffected by early exposure to electrical stimulation in comparison to cell cover formation [11]. The effect of stimulation has been shown to account for 10% to 20% of the total drop in impedance from 2 weeks to 10 weeks postoperatively, but for only 5% from 1.5 to 5 years after the implantation in patients receiving CIs [5–7]. This could be explained by the further development of fibrosis at a later stage post-implantation which may not be affected by electrical stimulation to a great extent.

#### Evolution of apical electrode impedance

The reason why impedance reached a peak at 1 week and dropped thereafter in the apical electrodes is unclear (Figs 2 and 3). Previous studies have shown that the impedance at basal, mid-portion and apical areas increases gradually for 2 weeks postoperatively before activation [20, 21], which may imply that the unique finding of apical electrode impedance in our series was associated with early activation. One possibility is that acute inflammation caused by insertion may have persisted for several days, and that cell cover formation was likely due to immune cell adhesion and protein adsorption. The acute immune reaction then subsided gradually as the electrochemical cleaning effect of the cell cover persisted after 1 day of activation. The peak impedance values in the apical electrodes at 1 week may reflect a plateau of the inflammation process, and the significant drop in CH 20, CH 21 and CH22 between 1 week and 4 weeks in our series may imply that inflammation subsided during this period (Table 2).

#### Evolution of basal and mid-portion electrode impedance

In contrast, the influence of the fibrotic process at the basal and mid-portion electrodes may have been stronger than the weakening immune reaction. A more pronounced fibrotic reaction at the basal part of the electrode compared to the apical part has been reported in several studies [20, 25, 26]. This may explain the gradual increase in impedance at the basal and mid-portion electrodes after 1 day of activation in our series (Figs 2 and 3). In addition, a significant increase in impedance in CH 1, CH 6, CH 7, CH 9, CH 10, and CH 11 was noted between 1 week and 4 weeks in our series (Table 2). Except for CH 1 at cochleostomy, CH 6, CH 7, CH 9, CH 10, and CH 11 were located over the first cochlear turn, where the lateral wall of the cochlea can be easily harmed during insertion [8, 27]. Our findings are compatible with a previous study in which the concentration of fibrosis and new bone formation at the cochleostomy and ascending limb of the basal turn corresponded to sites of trauma to the lateral cochlear wall [28]. Furthermore, this finding also indicates that the fibrotic process was significant from 1 week to 4 weeks postoperatively.

### Stable impedance from 4 weeks to 8 weeks

There were no significant differences in impedance in any of the channels between 4 weeks and 8 weeks in our series, demonstrating that impedance was relatively stable 4 weeks after surgery (Table 2). This suggests a delicate balance between cell cover formation, a divergence effect with electrical stimulation, and pro- and anti-fibrotic cytokines [29]. The further evolution of impedance after 2 months has been reported in several studies with no significant changes in adults [8, 15, 18, 19].

### Limitations

We only investigated one implant system (Nucleus 24RECA implant system) in this study. Additional studies are required to investigate the detailed evolution of impedance after early activation with different implant systems [10, 30] to validate our results in devices with different designs. In addition, further studies are needed to investigate changes in maximum comfort and threshold levels [31] after early activation to better facilitate the mapping process in patients.

## Conclusions

The early activation of a CI has become increasingly common in recent years because of improvements in surgical techniques. This study is the first to report the evolution of impedance in all channels after 24 hours of activation. We found a significant drop in impedance at 1 day followed by a significant rise at 1 week, and diverse findings at different segments thereafter. The mechanism may be associated with the combined effect of dynamics among the interplay of cell cover formation, electrical stimulation, and fibrotic reaction.

## Supporting information

S1 File Monopolar 2 mode impedance values of all patients. (XLSX)
