# Correction: Evolution of impedance field telemetry after one day of activation in cochlear implant recipients

**DOI:** 10.1371/journal.pone.0175833

**Published:** 2017-04-10

**Authors:** Hao-Chun Hu, Joshua Kuang-Chao Chen, Chia-Mi Tsai, Hsing-Yi Chen, Tao-Hsin Tung, Lieber Po-Hung Li

There are errors in the Funding section. The correct funding information is as follows: This work was supported by Cheng Hsin General Hospital (105–11) of Taiwan. The funders had no role in study design, data collection and analysis, decision to publish, or preparation of the manuscript.
